# ROW1 maintains quiescent centre identity by confining *WOX5* expression to specific cells

**DOI:** 10.1038/ncomms7003

**Published:** 2015-01-29

**Authors:** Yuzhou Zhang, Yue Jiao, Zhaohui Liu, Yu-Xian Zhu

**Affiliations:** 1 State Key Laboratory of Protein and Plant Gene Research, College of Life Sciences, Peking University, Beijing 100871, China; 2 National Center for Plant Gene Research (Beijing), Beijing 100101, China

## Abstract

The quiescent centre (QC) in the *Arabidopsis* root apical meristem is essential for stem cell organization. Here we show that the loss of REPRESSOR OF WUSCHEL1 (ROW1), a PHD domain-containing protein, leads to QC failure, defects in cell differentiation and ectopic expression of *WUSCHEL-RELATED HOMEOBOX 5* (*WOX5*) in cells that normally express *ROW1*. The *wox5-1/row1-3* double mutants show similar phenotypes to *wox5-1* indicating that *WOX5* is epistatic to ROW1. ROW1 specifically binds trimethylated histone H3 lysine 4 (H3K4me3) in the *WOX5* promoter region to repress its transcription. QC expression of *ROW1* results in a *wox5-1*-like phenotype with undetectable *WOX5* transcripts. We propose that ROW1 is essential for QC maintenance and for stem cell niche development through the repression of *WOX5* in the proximal meristem.

## Abstract (web summary)

The quiescent centre controls stem cell differentiation at the root apical meristem. Here Zhang *et al*. propose that ROW1 maintains meristem cell identity by repressing expression of the *WOX5* transcription factor in the proximal meristem zone, thus confining its activity to the quiescent centre.

## Graphical abstract

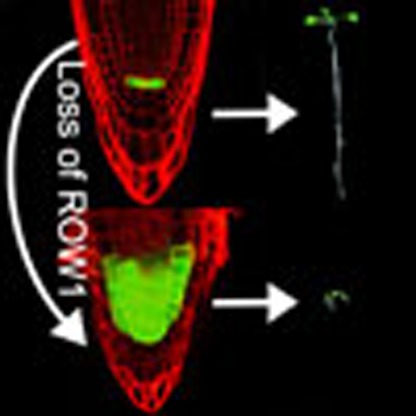

A plant seedling typically starts out with two meristems, which are situated at the tip of the shoot and the root^1^. The organizing centre (OC) and the quiescent centre (QC) play essential roles in maintaining stem cell populations within shoot and root meristems, respectively^2^,^3^. OC-specific *WUS* transcription is required for stem cell homeostasis in the shoot apical meristem (SAM)^4^,^5^. CLAVATA 3 (CLV3), a small peptide specifically expressed in the central zone immediately above the OC, represses *WUS* expression outside of the OC by binding to another small peptide CLV1 (ref. 6). Activation of CLV1 and related receptor kinases mediates the repression of *WUS* transcription through a signalling cascade that is not well understood^7^,^8^. On the other hand, OC-expressed *WUS* was found to migrate into the central zone to activate CLV3 transcription in a feedback loop^9^ that maintains the number of stem cells in the SAM. Likewise, the stem cell population in the root apical meristem is maintained by confining the expression of *WUSCHEL-RELATED HOMEOBOX 5* (*WOX5*), a homeobox transcription factor^10^,^11^ to the QC. The root stem cell niche is positioned in the apical–basal direction along an auxin gradient, which is produced by the polar localization of PIN-FORMED proteins^12^. Auxin regulates distal meristem (DM) cell differentiation by acting on *WOX5* (ref. 13). AUXIN RESISTANT 3 (AXR3), a member of the AUX/IAA family, is required for auxin signalling through the auxin response factors ARF10 and ARF16 (refs 13, 14). Recent work revealed that the WOX5–AXR3 feedback circuit is essential for the auxin-mediated DM differentiation in the *Arabidopsis* root^15^. To date, the mechanisms of QC-specific *WOX5* expression, QC identity maintenance and proximal meristem (PM) differentiation remain largely unknown.

The first discovered plant homeodomain (PHD)-containing protein with a canonical Cys4-His-Cys3 zinc finger motif was HAT3.1 (ref. 16), which regulates nuclear processes involving chromatin covalent modifications, especially histone H3 lysine 4 (H3K4) methylation^17^,^18^. Trimethylated H3K4 (H3K4me3) is proposed to regulate gene expression through its recognition by transcriptional activators^19^,^20^. Previous studies revealed that certain PHD domain-containing proteins bind to H3K4me3 to regulate target gene transcription via histone acetyl transferase activities or histone deacetylase chromatin-modifying complexes^20^,^21^,^22^. REPRESSOR OF WUS1 (ROW1) is a PHD-containing protein^23^ with two tandem BRCA1 C-terminal domains and a RING domain^24^. The BRCA1 C-terminal domains of ROW1 are important for phosphorylation-dependent protein–protein interactions^25^, and the RING domain is required for DNA repair^26^. Mutations in *ROW1* (formerly called *BARD1* (ref. 23)) cause severe SAM defects in *Arabidopsis* by releasing *WUS* expression from its normal confinement in the OC to the outermost cell layers^24^.

In this study, we show that ROW1 is required to maintain QC identity and stem cell niche development. We propose that it functions by suppressing *WOX5* expression in the PM of *Arabidopsis* root. We believe that it is the first key repressor that maintains both root apical meristem and SAM structures by interacting with *WUS* and *WOX5* independently.

## Results

### Root phenotypes of the *Arabidopsis row1-3* mutant

**Figure 1:**
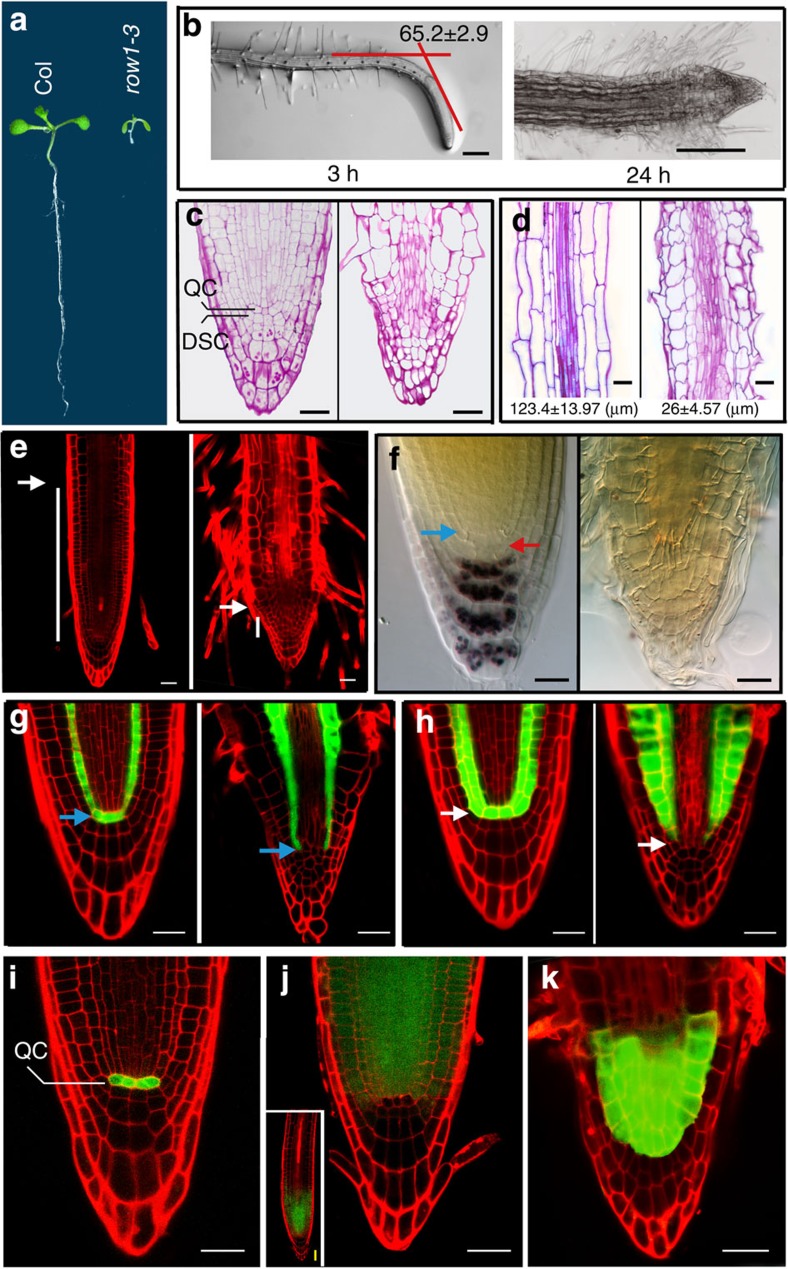
Phenotypic and expression pattern analysis in *row1-3* roots. (**a**) A 2-week-old wild-type *Arabidopsis* seedling (Col) showed normal root length (left), whereas a *row1-3* root (right) did not elongate. (**b**) Gravitropic responses in wild-type (left) and *row1-3* (right) roots. Degrees of bending (mean±s.e.) were calculated from 10 independent main roots of each type. (**c**) Median longitudinal semi-thin sections of 7-day-old wild-type (left) and *row1-3* (right) root tips stained with periodic acid–Schiff solution. (**d**) Cell length measurements from maturation zone in wild-type (wt) and *row1-3* roots. Root tips from 7-day-old seedlings were used for semi-thin section preparations; cell lengths (mean±s.e. in μm) were obtained from three seedlings of each type. (**e**) 7-day-old root tips of wild type (left) and *row1-3* (right). White bars indicate the size of PM regions. (**f**) Wild-type root tip accumulated starch granules in columella cells (left), whereas no starch granule was observed in *row1-3* root tips (right). Starch granules were stained with the Lugol’s solution and seen as aggregated black spots. Blue arrow, QC position; red arrow, DSC layer. (**g**) SCR::GFP expression showing normal QC identity (blue bars) in the wild-type (left) and a defective QC position in *row1-3* roots (right). (**h**) Expression pattern analysis of the GFP-enhancer trap line J0571 that show normal QC identity in the wild type (left) with a defective QC in *row1-3* roots (right). (**i**) WOX5::GFP signals were detected specifically in the QC in wild-type seedlings. (**j**) ROW1::GFP signals were detected above, but not in, the QC position in wild-type seedlings. Inset, a lower-magnification micrograph shows the whole root tip. (**k**) WOX5::GFP signals were detected in cells above the normal QC position in *row1-3* root. Scale bars, 20 μm in this figure.

Here we observed severe root architecture defects, including extremely short roots (Fig. 1a) and loss of gravitropic response (Fig. 1b) in the *row1-3* knockout mutant. Median longitudinal semi-thin sections showed no obvious QC identity, no distal root meristem (DSC) structure and no starch granules that indicates a defect in columella cell differentiation in the mutant (compare the wild type shown on the left of Fig. 1c to the mutant on the right). Sections prepared from the maturation zone, however, displayed a drastic difference in cell length between the two seedlings (Fig. 1d). Interestingly, the loss of gravitropism in *row1-3* was independent of auxin signalling, because both mutant and wild-type seedlings showed similar expression patterns of the synthetic auxin-responsive promoter reporter DR5::GFP after gravistimulation (Supplementary Fig. 1). In *row1-3* roots, root hair cells emerged very close to the root tip, indicating that its PM is defective and largely consumed (Fig. 1e, with a wild-type root shown on the left and *row1-3* on the right). The root tips of *row1-3* are devoid of starch granules as evidenced by lack of staining with Lugol’s solution (Fig. 1f), indicating a complete repression of columella cell differentiation. Furthermore, a green fluorescent protein (GFP)-fusion reporter of SCARECROW (SCR), normally expressed specifically in the QC and the entire endodermis (Fig. 1g, left)^27^, was absent from the QC in *row1-3* mutants (Fig. 1g, right), indicating the loss of the QC identity. Meanwhile, the GFP-enhancer trap line J0571, specifically expressed in the QC and cortex/endodermis in wild type (Fig. 1h, left)^27^,^28^, was also absent from *row1-3* (Fig. 1h, right). Both these experiments indicated the loss of the QC identity in the mutant.

### Identification of *WOX5* as a target of ROW1 in roots

Because *WUS* is not expressed in the *Arabidopsis* root (Supplementary Fig. 2a)^29^, we analysed the WOX transcription factor gene family for possible ROW1 targets (Supplementary Fig. 3). Quantitative reverse transcription-PCR (qRT–PCR) analysis identified *WOX5* as the most significantly (<0.001) upregulated *WOX* gene in *row1-3* roots (Supplementary Fig. 2b). In contrast, wild-type levels of *ROW1* transcript were found in the *wox5-1*-knockout mutant (Supplementary Fig. 2c). Further experiments, in corroboration with previous reports^30^,^31^, confirmed the exclusive expression of *WOX5* in the QC of wild-type roots (Fig. 1i), while *ROW1* is expressed in the PM above the QC (Fig. 1j). In *row1-3*, however, the *WOX5* expression zone is expanded into the multiple PM cell layers (Fig. 1k), suggesting that *WOX5* may be a target of ROW1.

### The *row1-3* root phenotype is complemented in *wox5-1*

**Figure 2:**
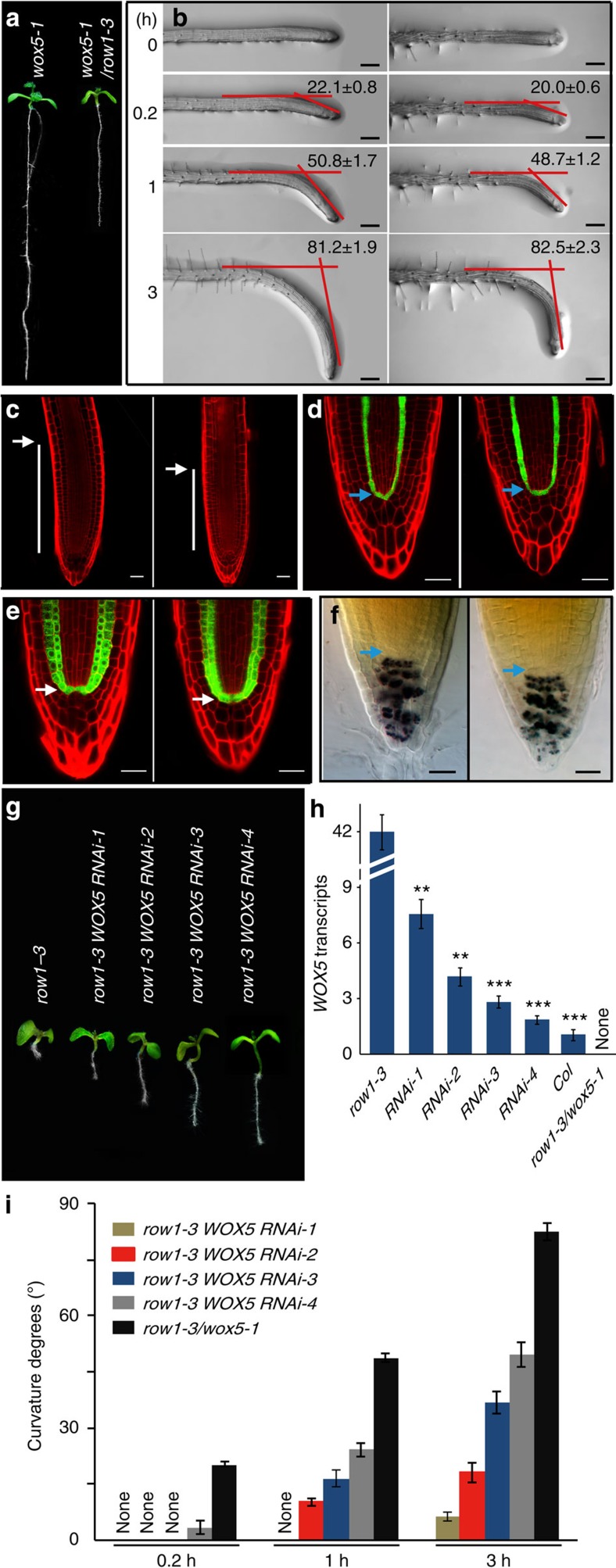
Complementation of the *row1-3* phenotype in the *wox5-1*/*row1-3* double mutant and in *row1-3 WOX5* RNAi lines. (**a**) Comparisons of root lengths at the 2-week-old stage. The *wox5-1* seedling has a root length similar to that of wild type; the *wox5-1*/*row1-3* double-mutant root was significantly elongated as compared with that of the *row1-3* single mutant (Fig. 1a). (**b**) Faster gravitropic responses were observed in roots of both *wox5-1* (left) and *wox5-1/row1-3* (right). Degrees of bending (mean±s.e.) were obtained from 10 seedlings. (**c**) 7-day-old root tips of *wox5-1* (left) and *wox5-1/row1-3* double mutant (right). White bars indicate the size of PM regions. (**d**) SCR::GFP expression indicating QC identity (blue arrow) in *wox5-1* (left) and also in the *wox5-1/row1-3* double mutant (right). (**e**) The GFP-enhancer trap line J0571 showing QC identity (white arrow) both in *wox5-1* (left) and in the *wox5-1/row1-3* double mutant (right). (**f**) Root tips of the *wox5-1/row1-3* double mutant (right) accumulated starch granules not only in columella cells but also in the DSC layer, identical to *wox5-1* (left). (**g**) Photographs of 7-day-old *row1-3 WOX5 RNAi-1*, *-2*, *-3* and *-4* in comparisons with *row1-3* and the *wox5-1/row1-3* double mutant. (**h**) qRT–PCR analysis of *WOX5* mRNA levels in *row1-3*, *row1-3 WOX5* RNAi lines, wild-type and *wox5-1/row1-3* roots, Error bars represent s.e. from three biological replicates. **, *** denotes *P*<0.01 or *P*<0.001, compared with the *row1-3*, respectively. (**i**) Comparisons of gravitropic responses of various *row1-3 WOX5* RNAi lines and *wox5-1/row1-3*. Degrees of bending (mean±s.e.) were calculated from 10 seedlings. Scale bars, 100 μm in (**b**) and 20 μm in (**c**–**f**).

Homozygous *wox5-1/row1-3* double-mutant seedlings showed substantial root elongation (Figs 1a and 2a) and PM restoration (Fig. 2c), compared with the *row1-3* mutant. The gravitropic curvature of *wox5-1/row1-3* is similar to *wox5-1* (Fig. 2b). QC identity and columella cell differentiation are both restored in the double mutant as indicated by SCR::GFP signal (Fig. 2d), J0571 marker (Fig. 2e) and Lugol’s solution staining (Fig. 2f). Similar to *wox5-1*, substantial amounts of starch granules accumulated in the DSC layer in *wox5-1/row1-3* roots (Fig. 2f), suggesting that these cells underwent premature differentiation.

### The *row1-3* root phenotype is restored in *WOX5 RNAi* lines

To examine more closely whether *WOX5* is a direct target of ROW1 function and that the defective root phenotype of *row1-3* is caused by ectopic *WOX5* expression, we used RNA interference (RNAi) to knockdown *WOX5* expression in the *row1-3* mutant. A series of RNAi lines, named *row1-3 WOX5 RNAi-1*, *-2*, *-3* and *-4*, were obtained based on their different root lengths, which correlated inversely with *WOX5* expression (Fig. 2g,h). Cells in the maturation zone of the RNAi lines were progressively longer than those in *row1-3* (Supplementary Fig. 4). The strongest RNAi line showed gravitropic responses close to the *wox5-1/row1-3* double mutant (Fig. 2i).

### ROW1 binds to H3K4me3 located at *WOX5* promoter region

**Figure 3:**
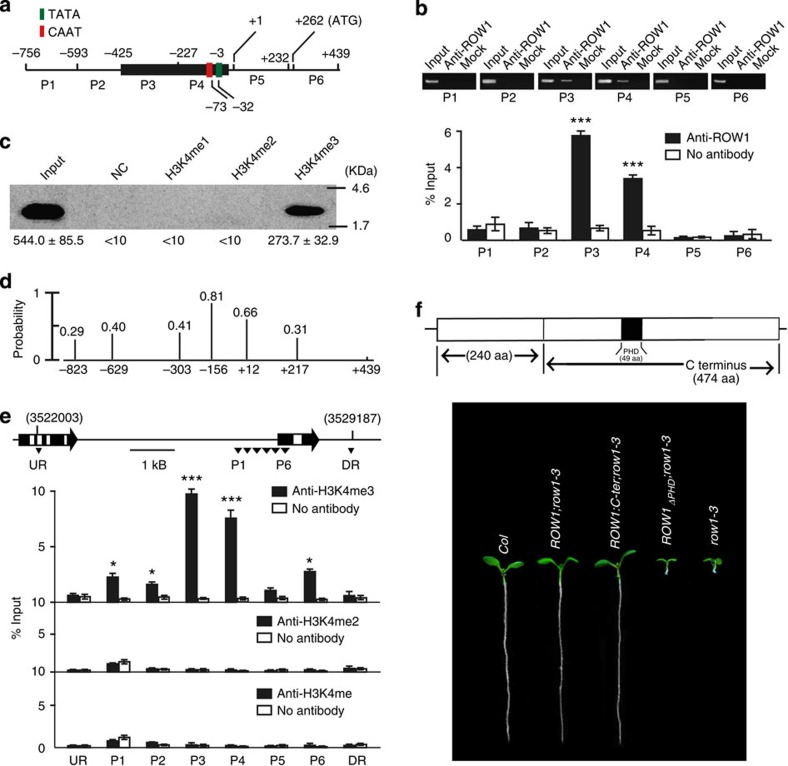
ROW1 specifically binds to H3K4me3 in the *WOX5* promoter region. (**a**) Schematic diagram showing the *WOX5* promoter region used in the ChIP assay. (**b**) ChIP analysis of different *WOX5* promoter regions using antibodies against ROW1. Upper panel, RT–PCR analysis. Input, 50 ng genomic DNA obtained from each respective promoter region; anti-ROW1, 10 μg of ROW1-specific antibodies were included in the reaction to precipitate the DNA; mock, negative control with no antibody added. Lower panel, qPCR analysis. Signal intensities were normalized relative to the input and were calculated from three independent experiments. (**c**) Purified PHD from ROW1 interacted only with biotinylated H3 peptides that were trimethylated at K4. Bound peptides were detected by western blot using biotin antibodies. (**d**) Theoretical analysis of nucleosome positioning along the *WOX5* promoter region using a previously reported computational model^32^. The *x* axis denotes *WOX5* chromosomal sequence from nucleotide −823 to +439 with the transcription initiation site set to +1. The *y* axis denotes the probability of predicted nucleosomes using the scale of 0.0–1.0. The exact positions of individual base pairs that has a >0.2 probability to initiate a nucleosome are shown as vertical black lines. (**e**) ChIP analysis of the same *WOX5* promoter regions using antibodies against H3K4me3, H3K4me2 or H3K4me. Upper panel, genomic location of *WOX5* is shown together with its direction of transcription. DR, downstream region; UR, upstream region. Lower panel, qPCR analysis. Signal intensities were normalized relative to the input and were calculated from three independent reactions. (**f**) ROW 1 or its C-terminal peptide (C-ter, 474 amino acids) rescued the *row1-3* phenotype, but a ROW1 construct with a 49-amino-acid deletion of the PHD failed to do so. Shown are 7-day-old seedlings of various genotypes carrying different constructs. Error bars represent s.e. from three biological replicates. *, *** denotes *P*<0.05 or *P*<0.001, compared with the negative control with no antibody added, respectively. Uncropped images of panels b and c are shown in Supplementary Fig. 14.

Next we studied the possible molecular interaction between ROW1 and *WOX5* using various fragments of the *WOX5* promoter depicted in Fig. 3a. The CAAT and TATA boxes were found –73 and –32 bp, respectively, upstream of the *WOX5* transcription initiation site as predicted by PlantCARE (http://bioinformatics.psb.ugent.be/webtools/plantcare/html/). We used a ROW1-specific polyclonal antibody generated against its C-terminal domain^24^ for chromatin immunoprecipitation (ChIP) analysis and found that this antibody specifically pulled down the proximal *WOX5* promoter region (fragments P3 and P4; Fig. 3b). *In vitro*-expressed and -purified His-ROW1-PHD specifically immunoprecipitated H3K4me3 but not H3K4me1 or H3K4me2 (Fig. 3c). This construct was also unable to pull down H3K9me3 and H3K27me3 (Supplementary Fig. 5). Theoretical nucleosome positioning analysis^32^ revealed that the proximal *WOX5* promoter region corresponding to P3 and P4 is likely packed into nucleosomes (Fig. 3d). The *WOX5* promoter with its P3 or P4 fragment deleted lost the ability to restore the *wox5-1* root phenotype (Supplementary Fig. 6a). At the molecular level, deletion of either one of the two fragments resulted in a non-functional promoter with no GFP expression in the wild-type background, whereas strong GFP signal is detected in plants carrying a full-length promoter or with the P1 fragment deleted (Supplementary Fig. 6b). ChIP using antibodies against histone H3 with different degrees of methylation at its K4 position showed that the P3 and P4 region was significantly enriched in H3K4me3 (Fig. 3e). P1, P2 and P6 regions displayed low-degree enrichment, indicating that *WOX5* may be under the regulation of multiple transcription factors, other than that of ROW1. The levels of H3K4me3 present in the *WOX5* proximal promoter region in both the *row1-3* and *wox5-1* mutants remained unchanged (Supplementary Fig. 7). A substantial reduction of the total amounts of H3K4me3 in *sdg2-1*, a major histone H3 lysine 4 trimethyltransferase in *Arabidopsis*^33^, is correlated with a significant *WOX5* upregulation (Supplementary Fig. 8). A deletion mutant of ROW1 that lacks the 49-amino-acid PHD lost all ability to restore the *row1-3* root phenotype as well as its binding to the *WOX5* promoter region (Fig. 3f and Supplementary Fig. 9), whereas a 474-amino-acid C-terminal peptide of ROW1 complemented the phenotype (Fig. 3f). Despite the fact that ROW1 binds effectively to the DNA fragment 4 of the *WUS* promoter, the gel shift assay showed it had no significant affinity to any of the DNA fragments of *WOX5* promoter used for ChIP assays (Supplementary Fig. 10).These results support the notion that ROW1 binds to histones in the proximal *WOX5* promoter region through recognition of H3K4me3 and that a functional PHD is essential for this interaction.

### QC expression of ROW1 represses *WOX5* transcription

**Figure 4:**
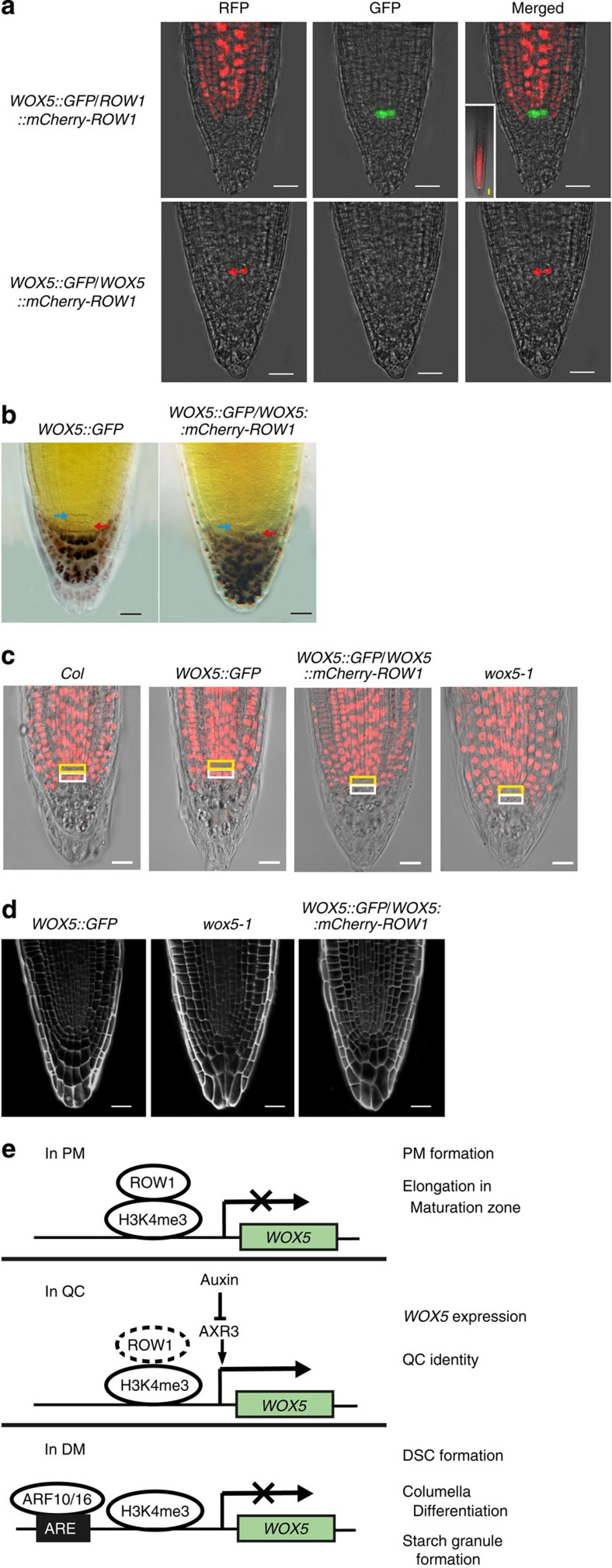
QC expression of *ROW1* reduced the amount of *WOX5* transcripts dramatically with a resultant loss of DSC. (**a**) GFP signals (green) were observed in QC and RFP signals (red) from mCherry-*ROW1* expression were observed in the PM from plants carrying both *WOX5::GFP* and *ROW1::mCherry-ROW1* constructs (upper panel), whereas only the RFP signals from mCherry-*ROW1* expression were detected from the plants carrying both *WOX5::GFP* and *WOX5::mCherry-ROW1* constructs (lower panel). (**b**) Root tips of *WOX5::GFP* plants showed normal QC (blue arrow) and DSC (red arrow, no starch granule) development, whereas starch granules appeared after staining with Lugol’s solution in the root tips of *WOX5::GFP/WOX5::mCherry-ROW1* plants. (**c**) EdU assay showing normal mitotic activity in wild-type (*col*) root tip, in root tips of wild-type plants that express the *WOX5::GFP* construct (*WOX5::GFP*), whereas no such mitotic activity was observed in the same cell layer in plants expressed the *WOX5::mCherry-ROW1* construct, similar to that of *wox5-1*. Yellow box, QC position; The white box below, DSC. (**d**) Three-dimensional reconstruction showing disrupted columella cell structure in 5-day-old root tips of both *wox5-1* and *WOX5::GFP/WOX5::mCherry-ROW1*. (**e**) A working model depicts the involvement of ROW1 during development of the *Arabidopsis* root stem cell niche. Scale bars, 20 μm in this figure.

To confirm that ROW1 represses *WOX5* expression *in vivo*, we ectopically expressed *ROW1* in the QC using the *WOX5* promoter. As a control for the study, ROW1::mCherry-ROW1 was introduced into wild-type *Arabidopsis* plants expressing the WOX5::GFP construct. As expected, red fluorescenct signal from mCherry was observed only in the PM, not in the QC, whereas green GFP fluorescence was observed strictly in the QC (Fig. 4a, upper panel). When expressed in *row1-3*, the ROW1::mCherry-ROW1 construct was able to complement the mutant phenotype (Supplementary Fig. 11). Only the red fluorescent protein (RFP) signals from mCherry-ROW1 expression were detected from the plants carrying both WOX5::GFP and WOX5::mCherry-ROW1 constructs. Although we expect that ectopic QC expression of mCherry-ROW1 will inhibit the *WOX5* promoter activity eventually, we propose that (1) a minimal amount of mCherry-ROW1 has to be present in the QC to repress the *WOX5* promoter activity and (2) that mCherry^34^ is more stable than the GFP, which originated from Dr5::GFP^35^, and that as a result, only the red mCherry RFP, and not the green GFP signal, was observed in the QC (Fig. 4a, lower panel). Inhibition of *WOX5* expression resulted in a *wox5-1*-like DM structure with substantial starch granule accumulation in the DSC layer (Figs 4b and 2f), suggesting that the DSCs in these plants underwent premature differentiation as well. This conclusion was verified by a 5-ethynyl-2′-deoxyuridine (EdU) incorporation assay of active cell division^36^. In wild-type *Arabidopsis* roots, regardless of a WOX5::GFP construct, cell division was observed in both PM and the DSC layer, not in the QC (Fig. 4c). However, no EdU incorporation was observed in the cell layer beneath the QC niche in plants expressing the WOX5::mCherry-ROW1 construct (Fig. 4c), indicating that QC expression of *WOX5* is essential for maintenance of DSC activity. Similar EdU incorporation pattern was observed in either *wox5-1* (Fig. 4c) or in *wox5-1*/*row1-3* double mutant (Supplementary Fig. 12) in which the DSC cell layer is known to undergo premature cell differentiation^10^. A regular and well-organized DM structure was found in the roots that expressed WOX5::GFP, whereas a disordered columella cell arrangement was observed in both *wox5-1* and wild-type plants expressing the WOX5::GFP and WOX5::mCherry-ROW1 constructs together (Fig. 4d). These data indicate that QC expression of *ROW1* inhibits *WOX5* transcription and results in premature differentiation of the DSCs and disruption of the regular columella cell structure in root tips. Taken together with previous publications^23^,^24^, we suggest that both *WUS* and *WOX5* are negatively regulated by the plant homologue of animal tumour suppressor-like gene *ROW1*.

## Discussion

As depicted in a working model in Fig. 4e, we propose that, in the wild-type *Arabidopsis* PM zone, ROW1 is bound to the H3K4me3 present on the *WOX5* promoter and represses its transcription to allow normal PM cell differentiation and elongation in the maturation zone. However, while we show that ROW1 is able to repress WOX5::GFP when ectopically expressed in the QC, deletion of the proposed ROW1-binding sites in the *WOX5* promoter did not induce *WOX5* expression in the PM (Supplementary Fig 6b). This suggests that the P3 and P4 promoter fragments are also necessary for *WOX5* activation and we cannot conclusively exclude the possibility that ROW1-mediated repression of *WOX5* in the PM is indirect.

In animals, the tumour suppressor protein ING2, which is also a PHD domain-containing protein, represses target gene transcription by binding to H3K4me3 histone markers^21^. In the QC, absence of ROW1 permits the expression of *WOX5* and thus maintains the QC identity. Auxin is the only molecule previously known to modulate QC function and distal stem cell differentiation by negatively regulating *WOX5* expression^13^,^14^,^15^. Significantly elevated *WOX5* expression in cells immediately above the DM in the root tips of *row1-3* may potentiate the diffusion of this small polypeptide, as was previously postulated^37^, to nullify ARF10/16 functions that prevented normal DM differentiation. We thus conclude that ROW1 is essential for the development of the whole stem cell niche in *Arabidopsis* roots by confining *WOX5* expression specifically to within the QC. Also, in the wild-type background, WOX5::GFP signals disappeared after 3 days of auxin treatment (Supplementary Fig. 13a), whereas no such repression for ROW1::GFP in the wild type and WOX5::GFP in the *row1-3* background were observed after the same treatment (Supplementary Fig. 13b,c), indicating that ROW1 may regulate *WOX5* expression downstream of auxin signalling. ROW1 may be the first reported key repressor that maintains both the SAM^23^,^24^ and the root apical meristem identity by interacting with two different master regulators of *Arabidopsis* stem cell development.

## Methods

### Plant lines and growth conditions

The *Arabidopsis* mutant lines with disrupted *ROW1* and *WOX5* are T-DNA insertion alleles obtained from the SALK collections (Arabidopsis Biological Resource Center, USA; http://signal.salk.edu): *row1-3*, SALK_003498 and *wox5-1*, SALK_038262. Seeds were surface sterilized with 0.1% HgCl_2_, germinated on Murashige and Skoog (MS) medium for 2 weeks, transferred to soil and grown in an Intellus control system (Percival) with a 16/8-h light/dark cycle at 22 °C in 70% humidity^38^. For microscopic analyses of gravitropism, seedlings grown in 1/2 MS medium in petri dishes were gravistimulated by rotating the stage 135° for the specified amount of time before imaging. Degrees of bending (mean±s.e.) were calculated from 10 independent main roots of each type.

### Plant crosses

Pollen collected from *wox5-1* plants was used to pollinate heterozygous *row1-3* plants to produce the homozygous *wox5-1/row1-3* double mutant. Transgenic plants carrying *WOX5::GFP* in the wild-type background^31^ were used for crossing with heterozygous *row1-3*. Plants that were homozygous for the *row1-3* mutation were used to search for the GFP marker in the F2 population. All analyses were performed using seedlings from the F3 generation. The transgenic *SCR::GFP* line (CS3999) and J0571 line (CS9094) were obtained from the SALK collections. *SCR::GFP* and J0571 marker lines were crossed to homozygous *wox5-1*, heterozygous *wox5-1/row1-3* and to heterozygous *row1-3* plants. In all analyses, parental lines were used as the controls.

### Vector construction and plant transformation

A *ROW1::GFP* line was obtained by transforming wild-type *Arabidopsis* plants with a vector in which the GFP expression was driven by the 1.9-kb *ROW1* promoter. The plasmid for *WOX5* RNAi was generated by cloning a 367-bp *WOX5* fragment from the 3′-transcribed region into the pB7GWIWG2 vector to create a double-stranded *WOX5* RNAi cassette driven by the constitutive cauliflower mosaic virus 35S promoter. This construct was then transformed into heterozygous *row1-3* plants to produce homozygous seedlings in the next generation. For genetic complementation of the *row1-3* phenotype, a 6.1-kb genomic DNA fragment that encompassed the entire *ROW1* (At1g04020) coding region plus 1.9 kb of the 5′ upstream sequence and 0.7 kb of downstream flanking sequence was cloned into pCAMBIA1305 using the primers described in Supplementary Table 1. This construct was then transformed into the heterozygous *row1-3 Arabidopsis* plants. The fusion ROW1 protein that lacked the PHD (residues 403–451) or C terminus (residues 241–714) was cloned into pCAMBIA3301 that contained the same *ROW1* promoter and downstream flanking sequences. Transgenic lines were selected by antibiotic resistance, genomic PCR and also by co-segregation studies that searched for single-copy insertion events into the *row1-3* homozygous background. We used 7-day-old seedlings of various genotypes carrying different constructs for phenotype analysis. The mCherry^34^ coding sequence was fused in-frame to the *ROW1* cDNA, and the resulting fusion was inserted into pQG110 carrying a 4.8 kb *WOX5* promoter or the 1.9 kb *ROW1* promoter, for transformation into the WOX5::GFP *Arabidopsis* line. Transgenic *Arabidopsis* plants were generated by the floral dip method and were selected on solid half-strength MS medium plates containing 50 mg ml^−1^ of the appropriate antibiotics.

### RNA extraction and qRT–PCR

Root and shoot apices were harvested from 7-day-old wild-type, *row1-3 wox5-1 Arabidopsis* seedlings. RNA extraction and qRT–PCR were performed as reported^24^. Seedlings (100 mg) were frozen in liquid nitrogen for RNA extraction with the RNeasy mini kit (Qiagen). Complementary DNA was synthesized from 5 μg of total RNA using reverse transcriptase (Fermentas), and the housekeeping gene *UBQ5* was used as the internal control. We used triplicate independent plant samples for all PCR analyses with the primers shown in Supplementary Table 1. We used the CT value method to quantify the relative amount of target gene transcripts as reported^24^. The relative value was calculated by the equation *Y*=1.8^Δ*C*t^ (Δ*C*t is the differences of *C*t between the target products and the control *UBQ5* products). Statistical significance was evaluated by Student’s *t*-test.

### Sequence alignment

Multiple sequence alignment was performed using MAFFT^39^ and phylogenetic trees were constructed by the neighbour-joining method in MEGA5 (ref. 40).

### Starch staining

*Arabidopsis* roots (7-day old) were dipped in Lugol’s staining solution (Sigma-Aldrich) for 60 min, washed with distilled water and then observed under a differential interference contrast microscope (Leica DMRE).

### Histological analysis

To prepare semi-thin sections, root tips were stained in 1% (w/v) periodic acid solution containing Schiff’s reagent and were fixed overnight in 2% (w/v) paraformaldehyde and 2.5% (w/v) glutaraldehyde in PBS, pH 7.2, at 4 °C. Specimens were then dehydrated in an ethanol series (30, 50, 70, 80, 90, 95 and 100%) and embedded in Spurr’s resin (Spi-Chem). The tissue was mounted in double-distilled H_2_O and sectioned at a thickness of 2 μm on a Leica RM 2265 microtome (Leica). Sections were observed under bright-field optics using a Leica DMRE microscope and cell lengths were measured by SPOT 4.6 Advanced software (Diagnostic Instrument, USA).

### ChIP assays

ChIP was performed as described^41^ using 7-day-old plants. For immunoprecipitation, 10 μg of commercial polyclonal antibodies against H3K4me3 (07-473), H3K4me2 (07-030) or H3K4me (07-436, Upstate/Millipore) or 10 μg of ROW1 polyclonal antibody^24^, were incubated in PBS solution with Protein A-agarose, in the presence of 1 mg chromatin extracts. One μg of immunoprecipitated DNA was used for each PCR assay. As negative controls, we performed the ChIP experiments using protein A-agarose without antibody. Relative enrichment of associated DNA fragments was analysed by qPCR. All oligonucleotide sequences used for target DNA detection and quantification in ChIP experiments are shown in Supplementary Table 1.

### EdU assay

Root tips of germinating *Arabidopsis* seedlings were submerged in 1 μM EdU in half-strength MS medium for 24 h (ref. 36). They were then fixed for 30 min at room temperature in a 4% (w/v) formaldehyde solution in PBS with 0.1% (v/v) Triton-X-100. The fixative was washed away with PBS (three 10-min washes) and the root tip sections were incubated in an EdU detection cocktail (Invitrogen, Click-iT EdU Alexa Fluor 555 Imaging Kit) for 30 min followed by three10-min washes with PBS. The sections were mounted in VECTASHIELD H-1000 anti-fade solution (Vector Laboratories) before being visualized using 545- to 600-nm wavelengths for EdU under an LSM 710 NLO with Duoscan confocal microscope (Zeiss, Germany).

### Histone peptide-binding assays

Biotinylated histone peptides H3K4me1 (12-563), H3K4me2 (12-460) and H3K4me3 (12-564) were bought from Upstate/Millipore, and H3K9me3 and H3K27me3 were provided described previously^42^. For the peptide-binding assay, each peptide (1 μg) was incubated with His-ROW1-PHD bound to Ni-NTA agarose beads in buffer containing 50 mM Tris-HCl, (pH 7.7), 300 mM NaCl and 0.1% (v/v) Nonidet P-40 for 1 h at 4 °C (ref. 20). The beads were washed five times for 30 min each in washing buffer at 4 °C, and the samples were separated by 15%Tris-tricine polyacrylamide gel electrophoresis and subjected to western blot analysis with antibody against biotin (Santa Cruz, sc-53179, 1:50)^43^.

### Confocal microscopy

For confocal microscopic analyses, 7-day-old seedlings grown in half-strength MS medium were stained with 10 μg ml^−1^ propidium iodide for 5 min (ref. 10), washed briefly in double-distilled and visualized at 600–640 nm for propidium iodide, 500–560 nm for GFP and 590–630 nm for mCherry RFP on the LSM 710 NLO with Duoscan confocal microscope. For three-dimensional reconstruction of wild type, *wox5-01* and *WOX5::GFP/WOX5::mCherry-ROW1* root tips, the cell walls were first visualized by staining with 10 μg ml^−1^ propidium iodide. A series of images was obtained by *z*-stack scanning and processed by ImarisX64 7.6.04 (Bitplane, Switzerland) to build the three-dimensional structure.

## Author contributions

Y.-X.Z. conceived and supervised the research. Y.-X.Z., Y.Z. and Y.J. designed the experiments. Y.Z. and Y.J. performed most of the experiments. Z.L. performed the experiments for Supplementary Fig. 4 and provided technical assistance to other experiments. Y.-X.Z. wrote the manuscript. All authors contributed to the manuscript and discussed the results extensively.

## Additional information

**How to cite this article:** Zhang, Y. *et al*. ROW1 maintains quiescent centre identity by confining *WOX5* expression to specific cells. *Nat. Commun.* 6:6003 doi: 10.1038/7003 (2015).

## Supplementary Material

Supplementary Information Supplementary Figures 1-14 and Supplementary Table 1.
