# Supplementary material for: ROW1 maintains quiescent centre identity by confining WOX5 expression to specific cells
Source: Nat Commun. 2015 Jan 29;6:6003. doi: 10.1038/ncomms7003 (PMC4316744; doi:10.1038/ncomms7003)
Supplement: Supplementary Information — Supplementary Figures 1-14 and Supplementary Table 1. [file ncomms7003-s1.pdf]

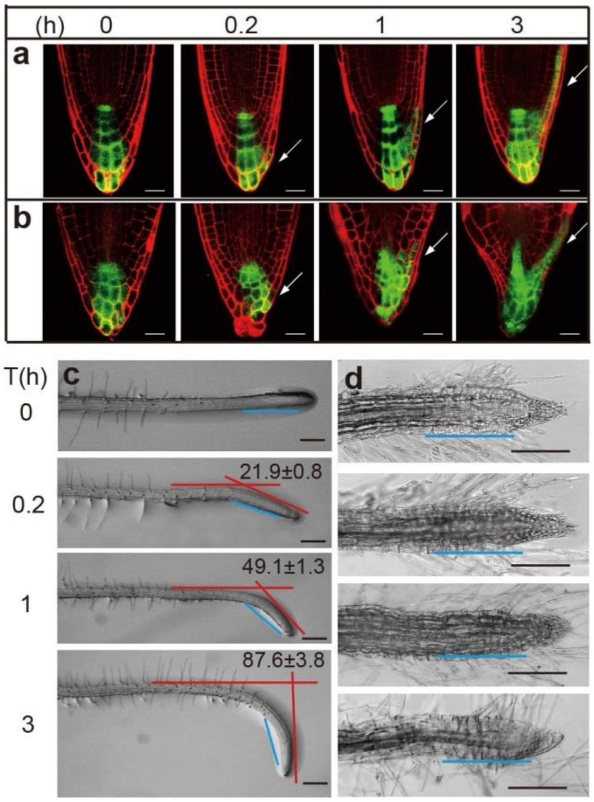

Supplementary Figure 1. ***row1-3*** **is defective in root gravitropic response but shows a normal asymmetrical redistribution of auxin. a,** Asymmetrical redistribution of DR5::GFP fluorescence signals in wild-type roots immediately after 0.2, 1 or 3 h of gravi-stimulation. **b,** Similar to wild type, the *row1-3* mutant showed DR5::GFP signal redistribution. **c,** Gravitropic responses in wild-type roots treated with 3 μM 1-naphthalene acetic acid (NAA), an auxin analogue, on the earthward side as indicated by the blue line. Bending angles (mean ± SE) were obtained from 10 seedlings. **d,** No gravitropic response was observed in *row1-3* roots after the same treatment. In **a** and **b**, bars = 20 μm; in **c** and **d**, bars = 100 μm.

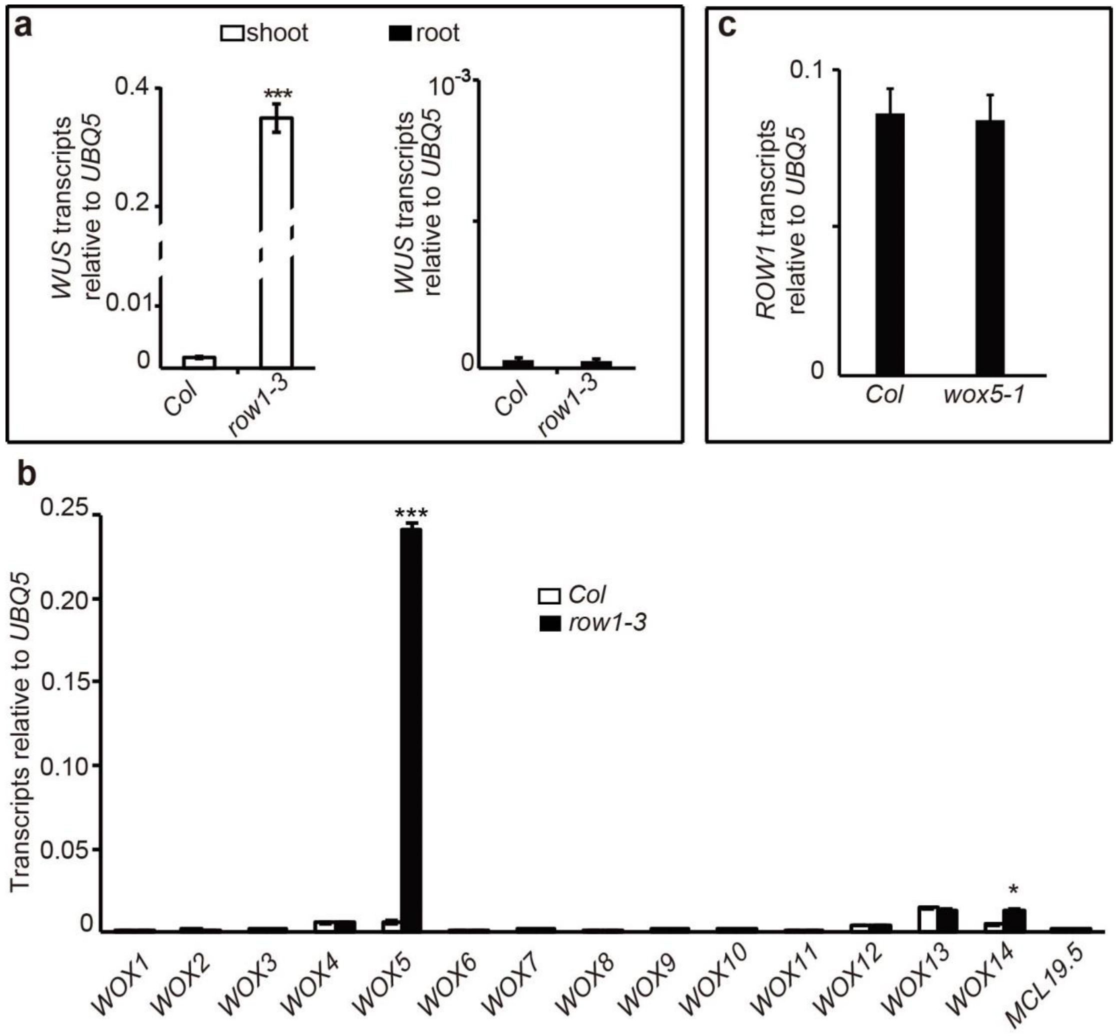

Supplementary Figure 2. Identification of *WOX5* as a potential target of ROW1 in *Arabidopsis* roots. a, Comparisons of *WUS* mRNA levels in wild-type and *row1-3* by QRT-PCR. **b,** QRT-PCR analysis of WOX subfamily gene expression in the roots of *row1-3* as compared with expression in wild-type roots. **c,** Wild-type *Arabidopsis* (Col-0) and *wox5-1* roots contained similar amounts of *ROW1* mRNA as quantified by QRT-PCR. Relative transcript levels (mean ± SE) were calculated from triplicate QRT-PCR experiments using independent RNA samples prepared from different batches of 7-d-old Arabidopsis seedlings. Constitutively expressed *UBQ5* was used as the internal standard for quantitative analysis. *P < 0.05 compared with wild-type; ***P < 0.001 compared with the wild-type sample.

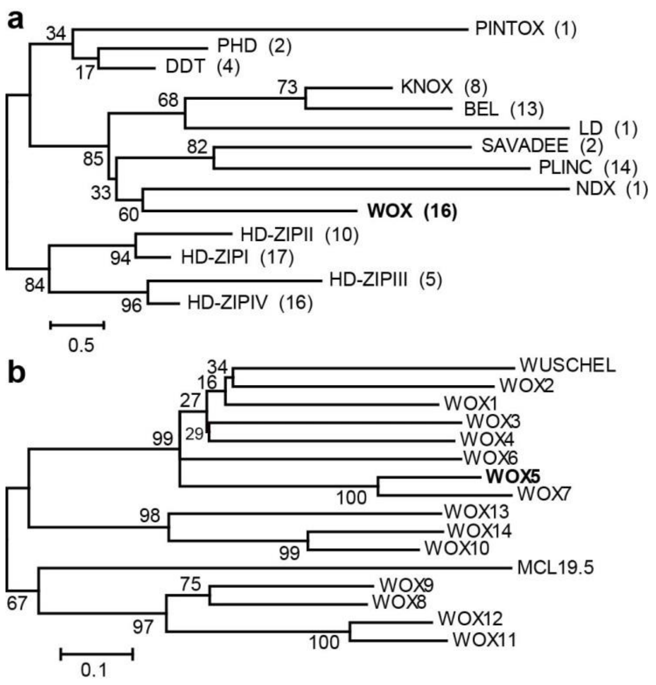

Supplementary Figure 3. Phylogenetic analysis of the Arabidopsis homeobox transcription factor families and the WOX subfamily. a, Phylogeny study of the homeobox transcription factor family by the neighbour-joining method using protein sequences for the analysis. Bootstrap values are shown on the branch points of the tree. The number of genes in each family is shown in parentheses. **b,** Detailed phylogeny study of the WOX subfamily.

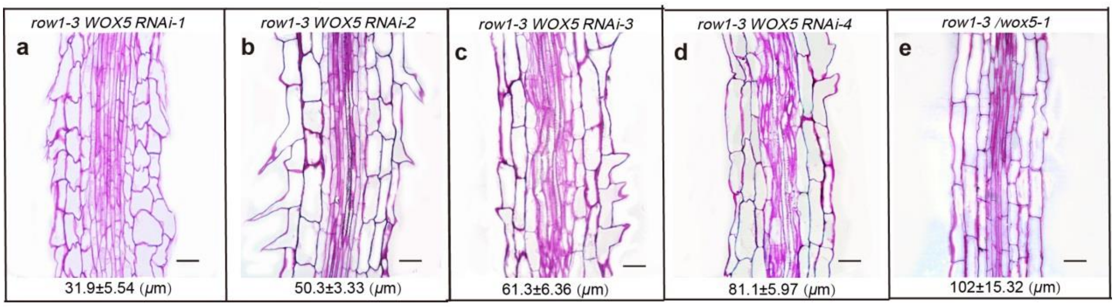

Supplementary Figure 4. Comparisons of cell lengths in maturation zones of various genotypes. (a–e) Median longitudinal semi-thin sections of 7-d-old *row1-3* WOX5 RNAi (**a–d**) lines and the *row1-3/wox5-1* double mutant (**e**) were stained with periodic acid–Schiff solution before being photographed. Measurements of cell lengths (mean ± SE) were obtained from 10 seedlings with two non-overlapping microscopic views from each. Bars = 20 μm in this figure.

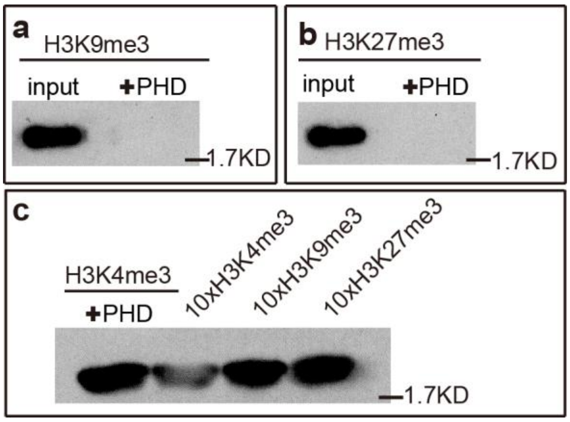

Supplementary Figure 5. ***In vitro*–expressed ROW1 PHD did not bind to H3K9me3 or H3K27me3, and H3K4me3 binding was competed only by unlabeled H3K4me3. a, b,** Binding to H3K9me3 (**a**) and H3K27me3 (**b**) was analysed. The input was 1 µg of biotinylated commercial H3K9me3 or H3K27me3, respectively. +PHD, 10 µg ROW1-PHD (67 aa from residues 388 to 454) was incubated in a HIS-tag column that contained the same amount of biotinylated H3K9me3 or H3K27me3, respectively. Bound peptides we detected by western blot using biotin antibodies as in Figure 3c. **c,** The binding between H3K4me3 and ROW1-PHD was successfully completed by 10 µg (10×) unlabeled H3K4me3, whereas no visible reduction was observed in the lanes with 10 µg (10×) unlabeled H3K9me3 or H3K27me3.

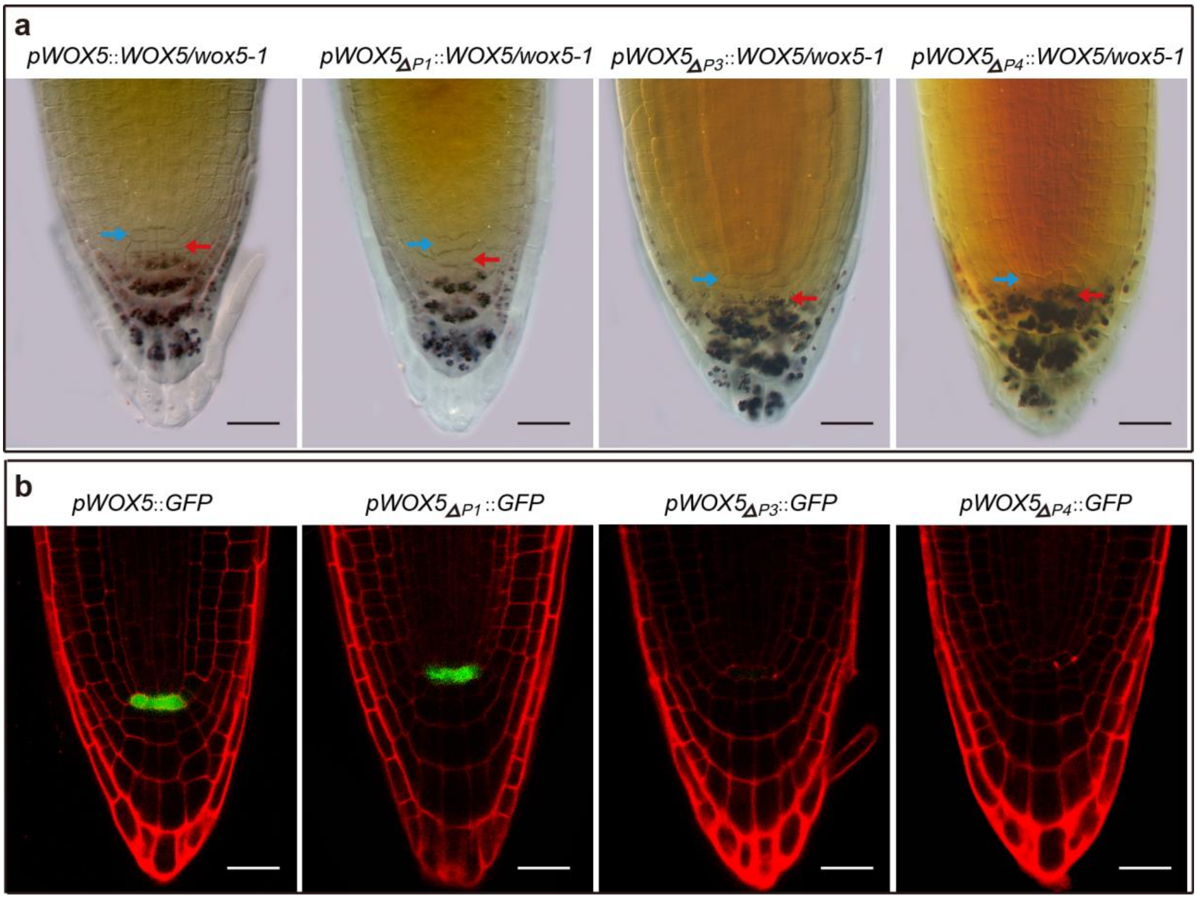

Supplementary Figure 6. Promoter deletion analysis revealed the importance of P3 and P4 promoter fragments in regulating *WOX5* expression. a, The *WOX5* promoter with its P3 or P4 fragment deleted lost the ability to restore the *wox5-1* root phenotype, whereas a full-length promoter or the promoter with P1 fragment deleted restored fully the *wox5-1* root phenotype. **b,** Deletion of P3 or P4 fragment resulted in a non-functional *WOX5* promoter with no GFP expression in wild-type background, whereas strong GFP signal is detected in plants carrying a full-length promoter or with the P1 fragment deleted. Bars = 20 µm in this figure.

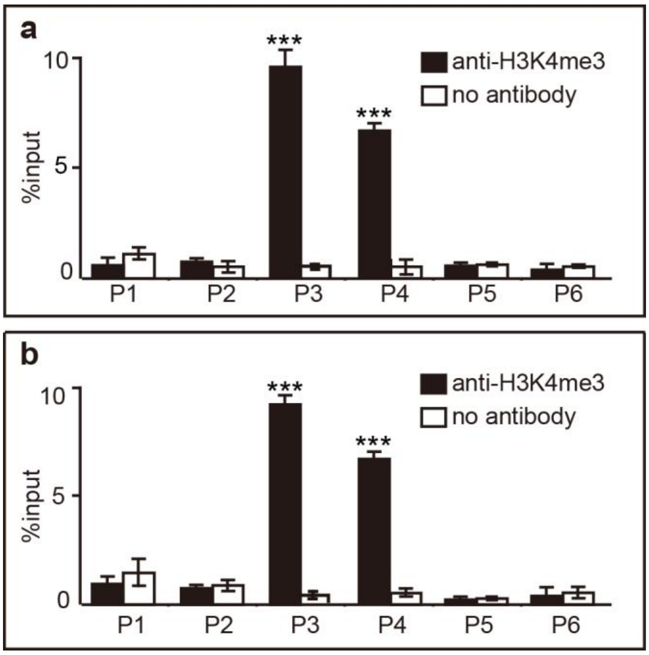

Supplementary Figure 7. Levels of H3K4me3 in *row1-3* and *wox5-1* mutants were identical to those in wild type. ChIP Q-PCR analysis of the same P3 and P4 *WOX5* promoter regions as reported in Figure 3e with antibodies against H3K4me3 using 7-d-old *row1-3* (**a**) and *wox5-1* (**b**) seedlings. Signal intensities were normalized relative to the input and were calculated from three independent ChIP Q-PCR experiments. Error bars represent SEs from three biological replicates. ***, denotes P < 0.001, compared to the negative control with no antibody added.

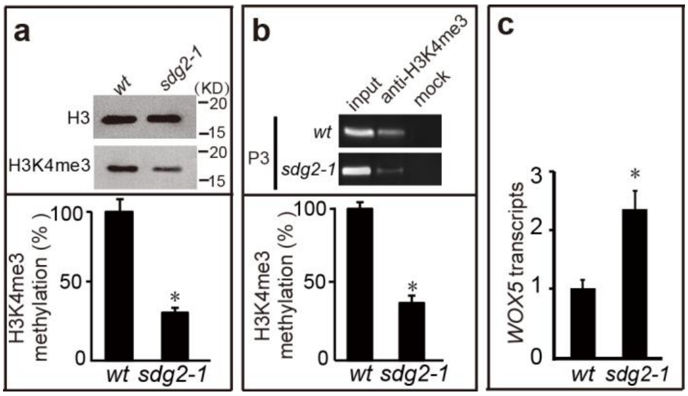

Supplementary Figure 8. The reduced H3K4me3 level in *sdg2-1* correlated with a significant increase in *WOX5* transcription. a, Western blotting analysis of H3K4 trimethylation showed a reduction in H3K4me3 levels in the *sdg2-1* mutant at the whole-genome level. H3 was used as the loading control. **b,** ChIP analysis of the P3 region using the same commercial antibodies against H3K4me3 as in Fig. 3e. The level of H3K4me3 at the *WOX5* promoter region was also significantly reduced in *sdg2-1* compared with that of the wild type. Statistical analyses obtained from three independent experiments are shown below a representative western blot in **a** and a representative ChIP assay in **b**. **c,** QRT-PCR analysis revealed that *WOX5* transcription was significantly upregulated in *sdg2-1*. The *WOX5* mRNA level in wild type root was arbitrarily set to 1. *P < 0.01. Statistics were obtained from three independent QRT-PCR experiments.

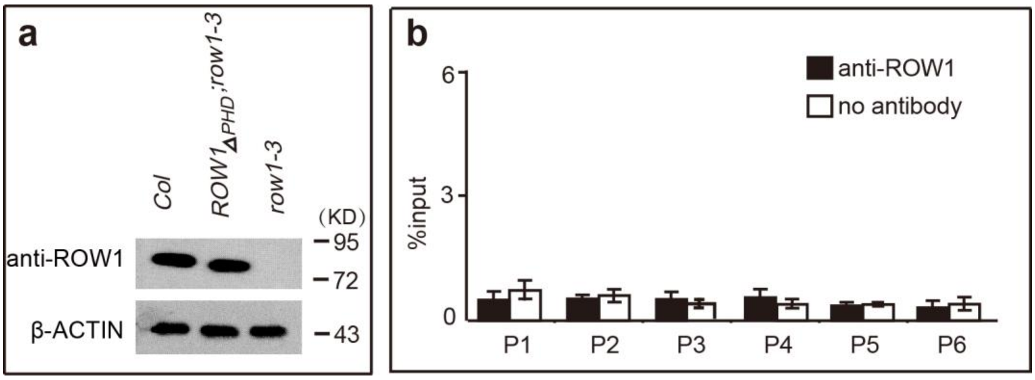

Supplementary Figure 9. A deletion mutant of ROW1 that lacks the PHD domain failed in binding to P3 and P4 regions of the *WOX5* promoter. a, Western blotting analysis of the stability of ROW1 protein lacking the PHD domain in *ROW1*$_{\triangle PHD}$;*row1-3* seedlings. **b,** ChIP Q-PCR analysis of *WOX5* promoter regions as reported in Figure 3b with antibodies against ROW1 using 7-d-old *ROW1*$_{\triangle PHD}$;*row1-3* seedlings.

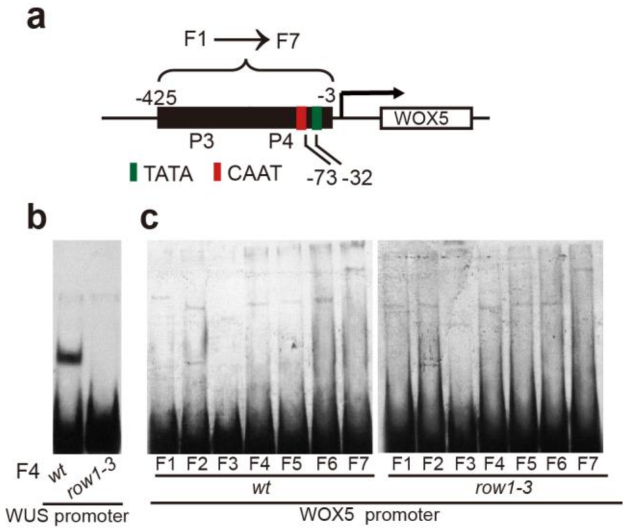

Supplementary Figure 10. ROW1 may regulate *WOX5* and *WUS* expression by different mechanisms. a, Schematic diagram showing the *WOX5* promoter region used for gel shift assays. Fragments: F1, -426 ~ 355; F2, -365 ~ -305; F3, -315 ~ -245; F4, -255 ~ -185 ; F5, -195 ~ -125; F6, -135 ~ -65; F7, -75 ~ -2 bp. **b,** ROW1 forms a specific protein-DNA complex on fragment 4 of the *WUS* promoter as previously reported[24]. **c,** Nuclear extracts prepared from wild-type plants can't form DNA-bound protein complex with the DNA fragments of the proximal *WOX5* promoter.

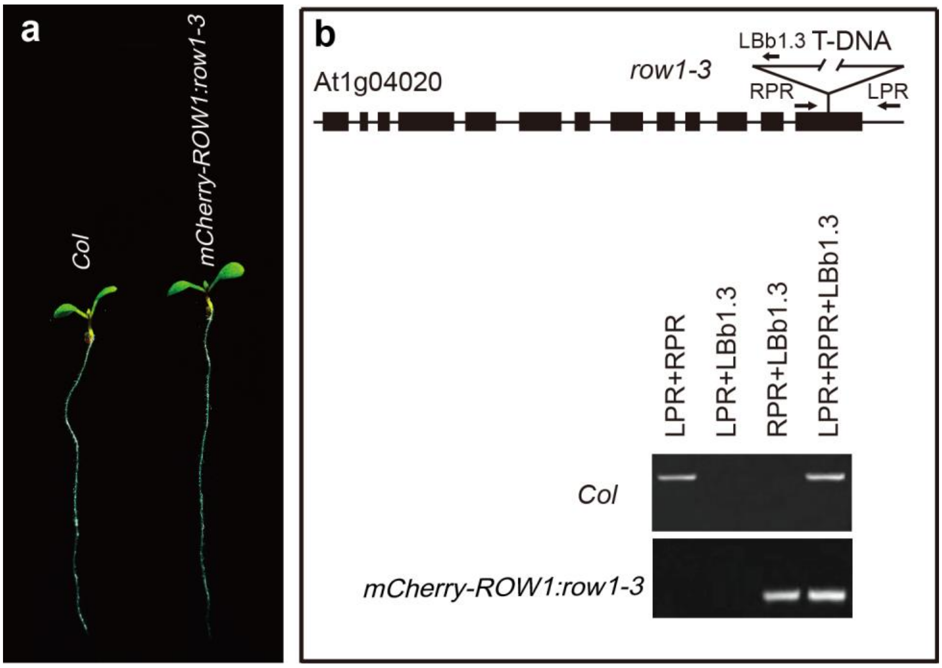

Supplementary Figure 11. The mCherry-ROW1 fusion protein has biological function *in vivo*. a, the mCherry-ROW1 construct is able to complement the *row1-3* phenotype. **b,** Genomic PCR analysis of homozygous *row1-3* mutant lines expressing the mCherry-ROW1 construct. RPR, complementary to the region downstream of the T-DNA; LPR, complementary to the region upstream of the T-DNA; LBb1.3, complementary to the left-most region of the T-DNA.

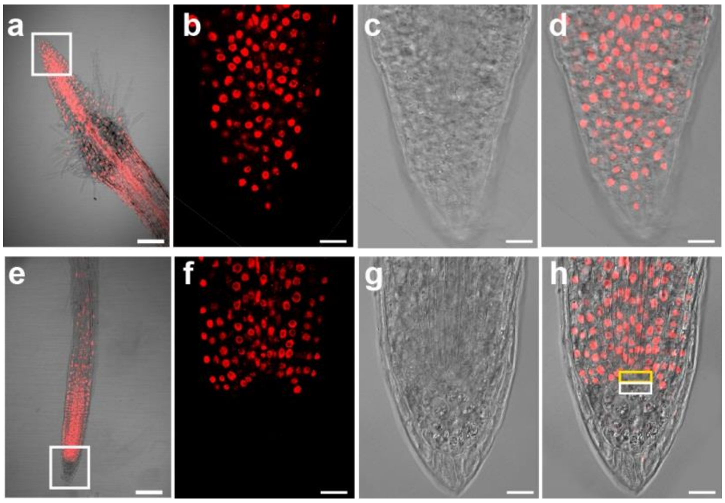

Supplementary Figure 12. EdU incorporation in the root tips of various *Arabidopsis* lines. Laser scanning confocal microscopy images of single optical sections of 4 μm (optical depth) in the median plane of *row1-3* (**a**–**d**) and *wox5-1*/*row1-3* double mutant (**e**–**h**) root tips treated with 1 μM EdU for 24 h in 1/2 MS medium. Differential interference contrast images were overlaid onto images of the red EdU signal. **a** and **e**, Low magnification, with scale bars = 100 μm, to show the whole root structure. **b** and **f**, The same analysis as above with higher magnification (scale bars = 20 μm) to show EdU incorporation in the QC and other cell types. **c** and **g**, Differential interference contrast images. **d** and **h**, Laser scanning confocal microscopy images merged with differential interference contrast images to identify the possible QC position (yellow box) and the DSC layer (the white box below).

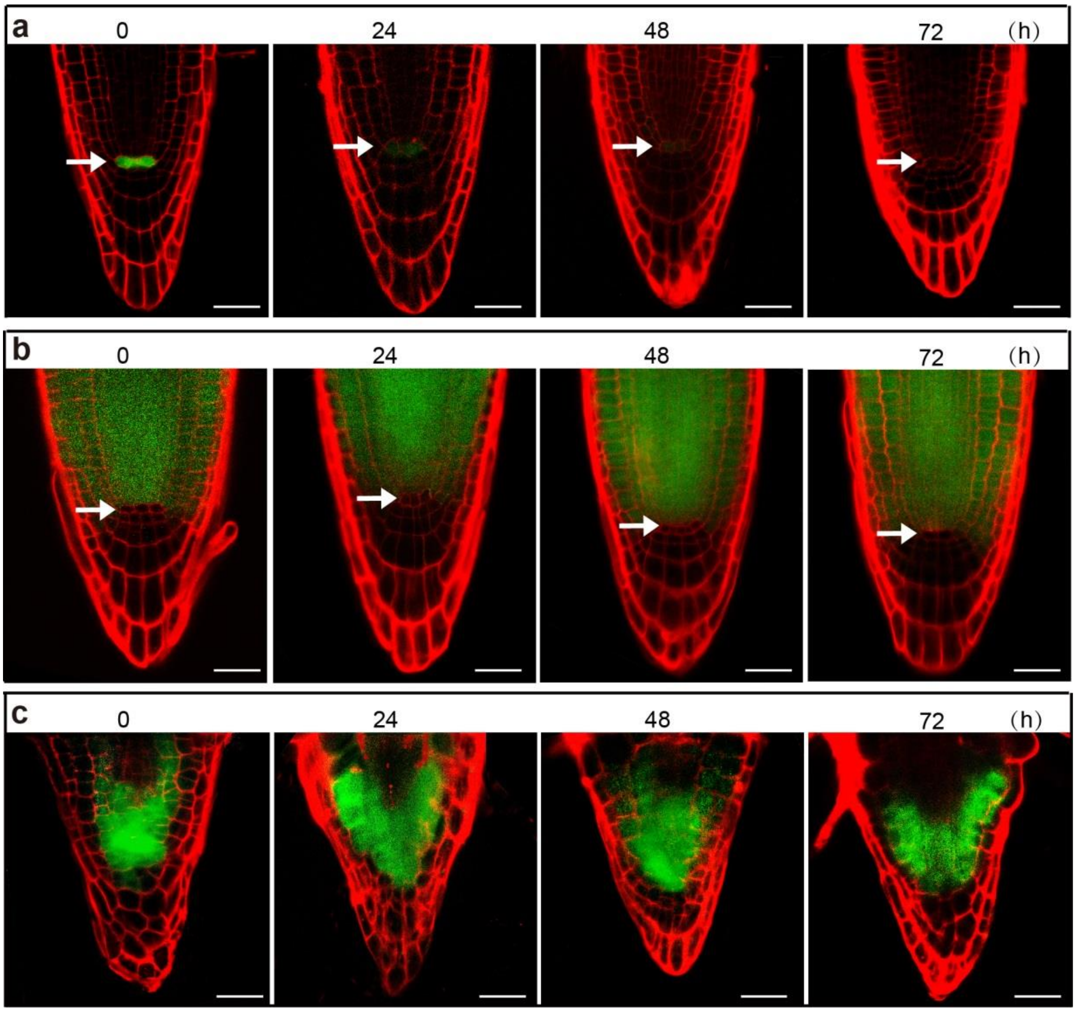

Supplementary Figure 13. ROW1 repression of *WOX5* may be downstream of the auxin signalling pathway. a, A gradual and substantial decrease in the WOX5::GFP signal in the QC after 24, 48 or 72 h of treatment with 5 μM NAA in wild type seedlings. **b,** ROW::GFP signal is not affected by the same 5 μM NAA treatment in wild type seedlings. **c,** No decrease in the WOX5::GFP signal intensity after the same period of NAA treatment in *row1-3* mutant seedlings. Scale bars = 20 μm.

Supplementary Figure 14. Full gel and blot scans relating to indicated figures.

Full gel for Fig. 3b

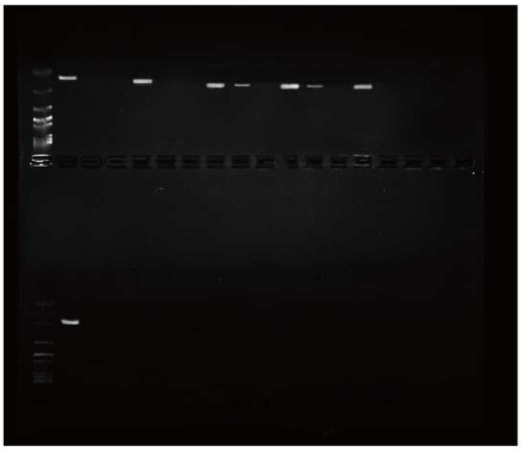

Full blot for Fig. 3c

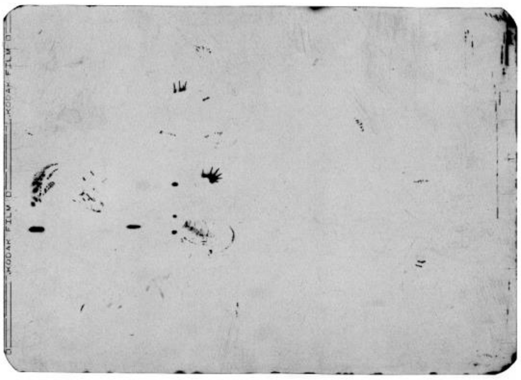

Full blot for Supplementary Fig. 5c

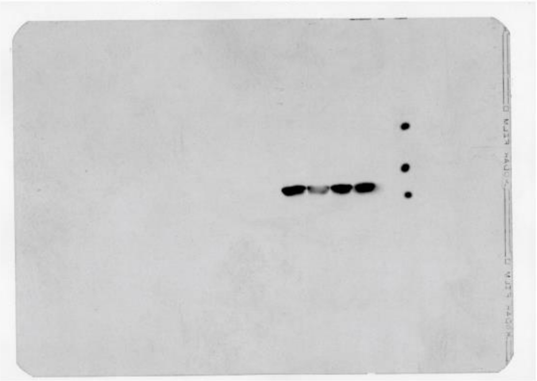

Full blot for Supplementary Fig. 5a (left) , b (right)

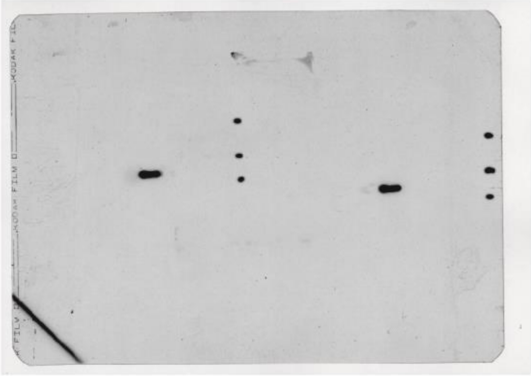

Full blots for Supplementary Fig. 8a

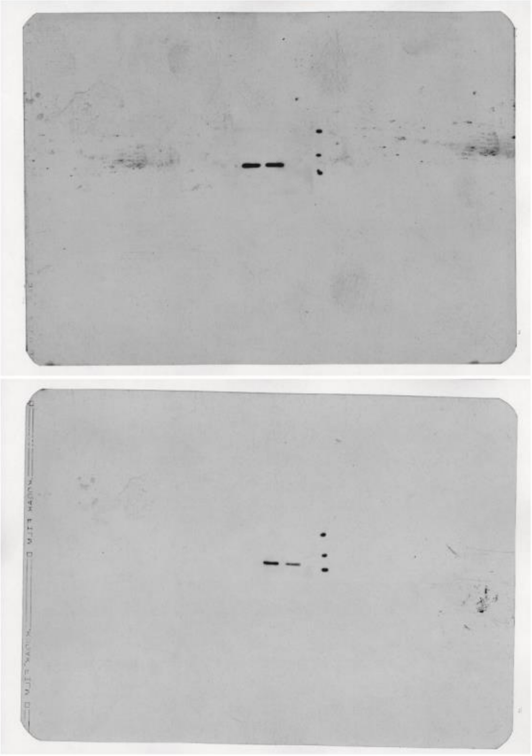

Full gels for Supplementary Fig. 8b

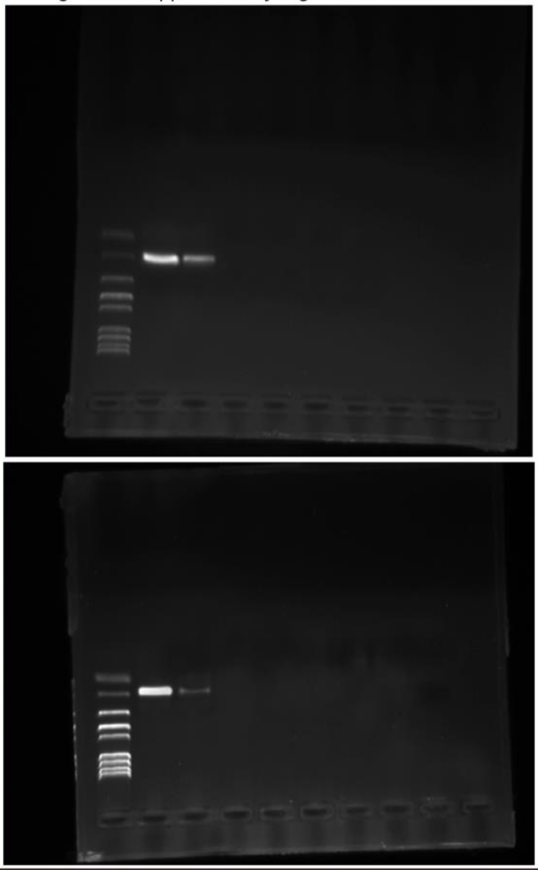

Full blots for Supplementary Fig. 9a

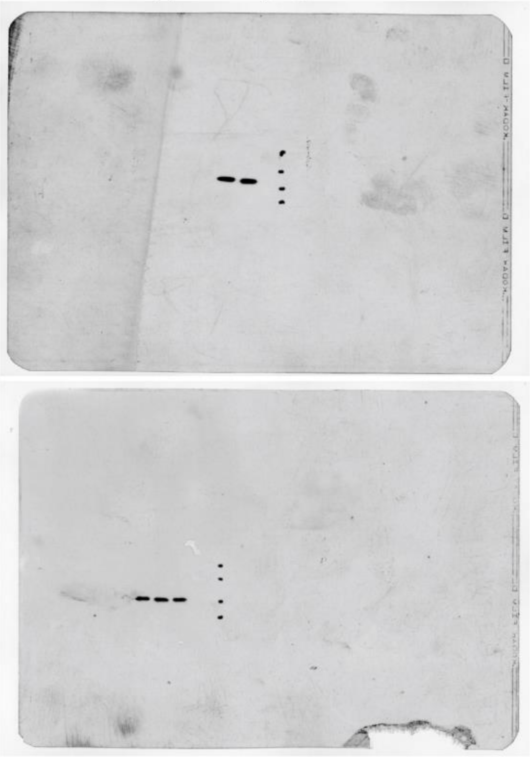

Full blot for Supplementary Fig. 10b

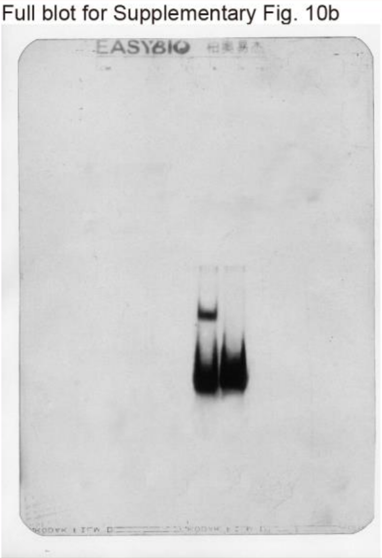

Full blot for Supplementary Fig. 10c

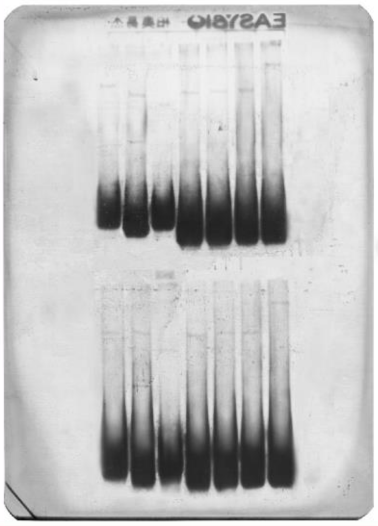

Full gels for Supplementary Fig. 11b

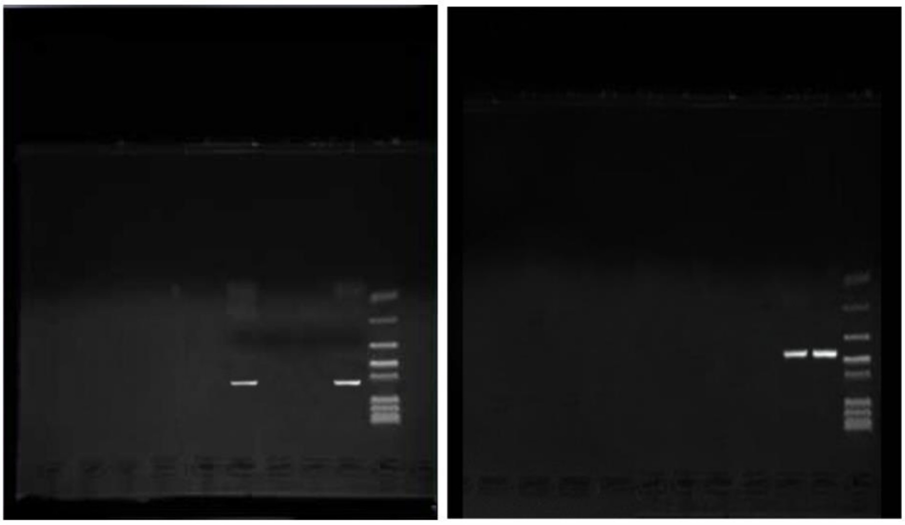

Supplementary Table 1 Primers used for QRT-PCR and other analyses

Gene	Primer sequences	Product length (bp)
QRT-PCR analysis		
WUS	5’-CGACGACGGAGCAAATCAAA-3’<br>5’-CATAGATCCATAGACATGGCT-3’	464
WOX1	5’-AACCACAAAGCCCGAGAACG-3’<br>5’-GCATCCGACCGAACATATCCAG-3’	168
WOX2	5’-GGCTTACTTCAATCGCCTCCTC-3’<br>5’-AGCCACCACTTGGAATCATCAC-3’	185
WOX3	5’-TTTGATTGCTGCTCTCATCCTTC-3’<br>5’-TACGATGAGTTTGGACCCGTG-3’	158
WOX4	5’-ACGACCACTGGTGTCTTTAATCC-3’<br>5’-TCTCTATCTCCAAGTTCTCAAATCC-3’	163
WOX5	5’-GTGGCAACAATAACGGAGG-3’<br>5’-TCTTGACAATCTTCTTCGCTT-3’	307
WOX6	5’-ACGACGGAACAGATCCAACAG-3’<br>5’-TTATGTGGTTTGATAATAGCACCAC-3’	152
WOX7	5’-AACACCGAGCACGGACCAG-3’<br>5’- CTTTCGCTGGTAGTTGATGACG-3’	218
WOX8	5’-TGGTAACGGAAGAAGGGATGG-3’<br>5’-TTAATAAACACCGTCATTCTCACC-3’	250
WOX9	5’-CTCTTGCCTTCTGCTTCTCACC-3’<br>5’-TCCGAATCTGCTCTGGCTTTG-3’	109
WOX10	5’-AGAACATTTACAAGGAAGGCAGTG-3’<br>5’-CCTAAATCAGGACTCGGGAACAG-3’	283

WOX11	5’-TTATTTGGTGGGTCATCTCAAGTTC-3’<br>5’-AGGAACACCTGAGGAATGCACC-3’	396
WOX12	5’-GTCGTCATCTCAAATCCCTTCC-3’<br>5’-AAACCAAACTCATCAGTGGGAAG-3’	339
WOX13	5’-ATAATGGGTTAGGGACAACAACAGC-3’<br>5’-CTTGTATTCAATCAGCCTGACATGC-3’	214
WOX14	5’-CGAAAGCAGCCTCAAACGAC-3’<br>5’-TCAATCCCTAAGTCAGGACTTGG-3’	143
MCL19.5	5’-GAACAAGAGGCGACATAGTGAA-3’<br>5’-TTTTCTTGGGTTTGTTCGGTGG-3’	112
UBQ5	5’-GGTGCTAAGAAGAGGAAGAAT-3’<br>5’-CTCCTTCTTTCTGGTAAACGT-3’	237
ROW1	5’-CCGCTAGGGTATCTGAGGC-3’<br>5’-CATAATCCCAACGGCATCT-3’	305
T-DNA		
LPR and<br>RPR	5’-TAGCTTCATCGGAATCTCTGC-3’<br>5’-CAAAAACCGCAAGACTCAGAG-3’	1098
LPW and<br>RPW	5’-ATCTCATAAACCATGCATCGG-3’<br>5’-TCGCTGGTTCCGATATACAAC-3’	905
LBb1.3	5’-ATTTTGCCGATTTCGGAAC-3’	
ChIP Q-PCR		
WOX5 P1	5’-ATATTATACATGTGTGTGGCGAACC-3’<br>5’-GTTGGTCGGCAAGTGTAGACAGG-3’	164
WOX5 P2	5’-CCTGTCTACACTTGCCGACCAAC-3’<br>5’-GCAAGTCCTAAACAAAGATTGTATGC-3’	191
WOX5 P3	5’-GCATACAATCTTTGTTTAGGACTTGC-3’<br>5’-AGAATAATCAGAAAGCCTTGGTGG-3’	232

WOX5 P4	5'-TACCACCAAGGCTTTCTGATTATTC-3'<br>5'-CCTAACCTATCTAGGCTTCTGTTCC-3'	244
WOX5 P5	5'-GGATAAAGAAAACGATCAAATCTGC-3'<br>5'-CGTTTTAGGGCCTGTGTATATATCC-3'	234
WOX5 P6	5'-ATACACAGGCCCTAAAACGTAAAAC-3'<br>5'-AACTGAGCTCCGTAGAGATCTTCTG-3'	226
WOX5 UR	5'-ATGCTTTCCTTCGTAGTAGGCTC-3'<br>5'-TTCAGCAAAACCTGTCAACAGTG-3'	196
WOX5 DR	5'-GATCGTTCACCCACTTGTCTTG-3'<br>5'-AAAATCAAGGCACCTGCGTAG-3'	150
Vector construction		
WOX5 RNAi	5'-GCGAAGAAGATTGTCAAGAGG-3'<br>5'-GACAACTTTTTGATAAACCATGC-3'	367
ROW1 promoter	5'- CCCAAGCTTCTCAGAAACAGGAAGGCCAAAC-3'<br>5'- AACTGCAGTGGTGATGTACAAAACCCAGATC-3'	1907
GFP	5'-TCCCCCGGGATGGTGAGCAAGGGCGAGGAGC-3'<br>5'-CGAGCTCTTACTTGTACAGCTCGTCCATG-3'	720
ROW1 genomic DNA	5'-ACATGCATGCCAAAAGGAAAATCCAGTGAGTT-3'<br>5'-ACATGCATGCCAAAAGGAAAATCCAGTGAGTT-3'	6054
WOX5 promoter	5'-AACTGCAGCGGTTTGTTTGACGAAGAGTA-3'<br>5'-CCCCCCGGGGTTCAGATGTAAAGTCCTCAACTG-3'	4801
ROW1 cDNA	5'-CGGGGTACCATGGCGGAATTTACTAACATGC-3'<br>5'-GTCGACTTAGCCAATCACAGGATGTAACTTG-3'	2145
mCherry	5'-CCCGGGATGGTGAGCAAGGGCGAGGAGGA-3'<br>5'-CGGGGTACCCTTGTACAGCTCGTCCATGC-3'	709
